# Conservative or surgical management of orbital schwannomas: a population-based case series

**DOI:** 10.1007/s00701-024-05899-1

**Published:** 2024-01-13

**Authors:** Victor Gabriel El-Hajj, Aman Singh, Cecilia Norin, Erik Edström, Elin Bohman, Adrian Elmi-Terander

**Affiliations:** 1 Department of Clinical Neuroscience, Karolinska Institutet, Stockholm, Sweden; 2 Division of Ophthalmology and Vision, St. Erik Eye Hospital, Stockholm, Sweden; 3 Capio Spine Center Stockholm, Löwenströmska Hospital, 194 02 Upplands-Väsby, Box 2074, Stockholm, Sweden; 4 Department of Surgical Sciences, Uppsala University, Uppsala, Sweden

**Keywords:** Schwannomas, Orbit, Orbital schwannomas, Surgery, Watchful waiting

## Abstract

**Abstract:**

**Introduction:**

Orbital schwannomas (OS) are rare occurrences with no more than 500 cases reported in the literature. The tumor’s potential to compromise the delicate neuro-ophthalmic structures within the orbit prompts surgical removal. Tumor removal is performed by ophthalmologists, often requiring a multidisciplinary surgical approach. The literature contains a very limited number of cases managed non-surgically. However, the inherent risks of orbital surgery warrant a comparison of the outcomes of conservative and surgical management strategies.

**Aims:**

To review the national Swedish experience with the management of orbital schwannomas.

**Methods:**

The study center is the primary Swedish referral center for the multidisciplinary management of orbital tumors, including schwannomas. During the period of 2005 to 2021, 16 patients with an OS diagnosis were managed at the center.

**Results:**

Four patients initially underwent surgery where gross total resection (GTR) was achieved in three (75%) and subtotal resection (STR) in one (25%) case. The remaining 12 patients, who had a low risk of neuro-ophthalmic impairment, were managed conservatively with radiological and clinical examinations at regular intervals. After an average follow-up of 17 months, surgery was performed in three of these cases (25%). No recurrences or tumor growths were detected on radiological follow-ups (mean 50 months), and all patients experienced postoperative improvement at clinical follow-up (mean 65 months). The remainder of the conservatively treated patients (*n*=9) experienced no clinical progression (mean 30 months). A slight radiological tumor progression was detected in one patient after 17 months.

**Conclusion:**

There were no differences in long-term outcome between patients who had been managed with early surgery and those operated later after an initially conservative management. Conservatively treated patients had minimal to no symptoms and remained clinically stable throughout the follow-up period. Based on these findings, conservative management may successfully be adopted in cases with mild symptoms, no signs of compressive optic neuropathy and low risk of neuro-ophthalmic impairment. Conversion to surgical management is indicated upon clinical deterioration or tumor growth. Based on the findings of this study a decision tree for the management of orbital schwannomas is suggested.

**Supplementary Information:**

The online version contains supplementary material available at 10.1007/s00701-024-05899-1.

## Introduction

Schwannomas are peripheral nerve sheath tumors (PNST) found in cranial and spinal nerves throughout the body. Schwannomas of the orbit are rare, and there are less than 500 cases reported in the literature [16]. It is estimated that these tumors constitute only about 1% of orbital tumors [9]. They are typically benign, slow growing, and encapsulated tumors that occur without any predilection for sex or age [16]. The growth of these tumors has been associated to functional and/or aesthetic morbidity and malignant transformation seems to be exceedingly rare [8]. Symptoms and clinical signs vary depending on the size and location of the tumor and may include a palpable mass, bulb dislocation, ptosis, optic neuropathy, and diplopia.

Management of these tumors is challenging and often requires a multidisciplinary collaboration that involves ophthalmologists, neurosurgeons, otolaryngologists, and maxillofacial surgeons. When possible, surgery with gross total resection (GTR) is the treatment of choice [5]. The orbit is a confined space delimited by bone, containing delicate neuro-ophthalmic structures and with a limited capacity to expand [15]. In the context of orbital schwannomas, curative approaches are often pursued to prevent tumor growth and subsequent compression and injury to intra-orbital structures [19]. The majority of cases in the literature have been offered surgery and most of the remaining have been treated with radiation therapy or stereotactic radiosurgery [12, 16]. Nevertheless, the surgical risks inherent to the complexity of the neurovascular structures of the orbit are not negligible [2, 6, 10, 18] and warrant further investigation of the outcomes of conservative management. Recently, three separate reports have questioned the validity of an aggressive treatment strategy and advocate watchful waiting in selected cases [3, 7, 16]. In addition, increased use, availability, and sensitivity of diagnostic imaging makes incidental findings of orbital tumors more common, which also indicates a need for conservative approaches.

In brief, the natural course of OS has been poorly studied and support for conservative management is consequently lacking. The aim of this study was to evaluate the national Swedish experience of surgical and conservative management of OS.

## Methods

### Admission routine

The study center is the primary Swedish referral center for multidisciplinary management of orbital tumors, including schwannomas. During the period of 2005 to 2021, 16 patients with a new diagnosis of OS were managed. Other neurosurgical and neuro-ophthalmological centers in Sweden were contacted to identify cases that may have been overlooked. One neurosurgical center reported having managed up to five cases during the study period. Unfortunately, no data on patients treated outside the authors’ institution could be retrieved.

Once a patient with a lesion suspicious of schwannoma is referred to the study center, a complete neuroophthalmological examination is performed. This includes additional imaging, automated perimetry, Optical Coherence Tomography (OCT) and fine-needle aspiration biopsy if deemed necessary. Management is then discussed at a multidisciplinary conference of ophthalmologists, neuro-ophthalmologists, radiologists, and in some cases neurosurgeons. Typically, a conservative management is considered for all patients with OS if the indications for early surgery are not met.

### Surgery and postoperative follow-up

Indications for early surgery are deformation of the globe or compression of the optic nerve leading to optic neuropathy. Relative indications for surgery may include impaired motility with double vision and cosmetic implications including proptosis, hyper- or hypoglobus, or ptosis with a palpable mass.

The surgical approach is determined by the surgeon’s preference and the tumor location, size, and extension. All procedures are performed with microsurgical techniques and under general anesthesia. Tumors extending into the orbital apex, or through the orbital fissure, or those causing severe thinning of adjacent bony structures are often operated jointly with a neurosurgeon. OS located in the superior part of the orbit can be accessed by an anterior orbitotomy through an eyelid crease incision. OS located posteriorly in the orbital apex, sometimes with skull base extensions, are often accessed transcranially via craniotomy.

In GTR, the nerve harboring the tumor is cut to allow complete removal of the tumor. In subtotal resection (STR), the tumor capsule is incised along the longitudinal axis of the nerve, and the schwannoma is excised leaving the capsule to retain the integrity of the nerve. This approach offers surgical plane separated from delicate structures surrounding the nerve, allowing safe tumor removal deep in the apex of the orbit.

A neuro-ophthalmic examination and MRI are performed at one-week post-surgery. If radicality is confirmed on MRI and the pathology report, no further follow-ups are required. However, in the case of STR, MRI is recommended at 3 months post-surgery and annually afterwards. In these cases, the follow-up is generally carried out at the primary referring clinic.

At follow-up, tumor growth was defined as the radiological growth of a tumor remnant following SRT, while tumor recurrence was defined as the reappearance of a tumor following GTR.

### Conservative management and follow-up

Conservative management is offered to patients with mild symptoms, no signs of compressive optic neuropathy and low-risk of neuro-ophthalmic impairment. This encompasses individuals incidentally discovered, and those reporting minimal subjective discomfort, proptosis, globe displacement, or ocular motility issues that do not interfere with everyday tasks, such as medical requirements for driving. Low-risk neuro-ophthalmic impairment is defined as cases where the tumor is not in the apex or direct contact with the optic nerve.

For these patients, radiological and ophthalmological examinations are performed at regular intervals. In patients where the tumor is in the orbital apex or located in the vicinity of the optic nerve automated perimetry and, in later years, OCT with measurements of the peripapillary retinal nerve fiber layer, macular ganglion cell layer thickness and macular ganglion cell inner plexiform layer is added. OCT with these measurements is a useful tool as they provide early signs of compressive optic neuropathy often preceding changes on automated perimetry [1, 17]. Additionally, patients are encouraged to seek care upon onset or worsening of symptoms. There is no consensus on the intervals or the optimal length of follow-up. In our practice, patients with stable tumors are examined twice a year during the first 2 years after the diagnosis and then once a year, until 5 years. However, longer follow-ups may be indicated in selected cases, including children. In patients with slowly growing tumors, MRI is performed twice a year until cessation of tumor growth or surgery. After the follow-up period patients are encouraged to seek care for any eye-related symptoms.

### Study setting

Patients included in this study were divided into 3 groups: patients with surgery as the primary management (group 1), patients converted to surgery after an initially conservative management (group 2), and patients conservatively treated throughout the entire follow-up period (group 3).

## Results

### Incidence

Considering the 16 documented cases of OS between the years of 2005 and 2021, the apparent incidence of OS in Sweden was estimated at 0.1 per million and year. However, the true incidence may be higher.

### Baseline characteristics

A total of 16 patients with OS, diagnosed in Sweden between 2005 and 2021, were included in this study (Table 1). Patients were aged between 8 and 74 years (median 52) at the time of diagnosis and 50% (*n* = 8) were female. Six patients (37%) were referred due to the incidental detection of an orbital mass during imaging for unrelated causes, such as dementia, stroke, or hydrocephalus. The remainder of the patients actively sought care for ophthalmic symptoms. Patients were referred to the study center under preliminary diagnoses, including OS, cavernous malformation, or dermoid cysts. A final diagnosis of OS was established in all cases, based on either histology and imaging (*n* = 11; 69%), or imaging alone (*n* = 5; 31%).

**Table 1:** Baseline characteristics of patients included in the study

ID	Sex, age, and chief complaint	Referral differential diagnoses	Tumor size (cm)	Location and extensions	Findings on MRI/CT	Basis of the final diagnosis	Management approach	Indications supporting the approach of choice
1	M, 15, hypoglobus	1- Schwannoma 2- Cavernous venous malformation	4.5x2.5x2.5	Intra- & extraconal, superior, medial, anterior, extends to the apex into the superior orbital fissure	Demarcated, heterogenous, lobulated, cystic, erosion of the roof and lateral wall of the orbit	Histology	Surgery	History of symptom worsening, and extent of the tumor
2	M, 57, incidental	1- Schwannoma 2- Dermoid cyst	2.0x1.5x1.0	Extraconal, superior, anterior	Demarcated, heterogenous, cystic, no erosion	Imaging	Observation	Incidental finding, with minimal associated symptoms
3	F, 44, pain and discomfort in the orbital region	Schwannoma	4.0x2.0x1.5	Extraconal, superior, lateral, anterior, extends to the apex and through the orbital roof	Demarcated, heterogenous, lobulated, cystic, erosion of the orbital roof	Histology	Biopsy and surgery	Tumor extension into the anterior fossa due to severe erosion of the orbital roof
4	F, 57, sudden vision loss	Schwannoma	1.4x0.9x0.8	Intraconal, posterior, with extension into the cavernous sinus	Demarcated, homogenous, cystic, erosion of the orbital fissure	Imaging	Observation	Rapid, spontaneous resolution of initial presenting symptoms
5	M, 26, incidental	1- Schwannoma 2- Dermoid cyst 3- Orbital venous varices	3.0x1.5x1.0	Extraconal, superior, medial, anterior, extends into the superior eyelid	Demarcated, homogenous, lobulated, cystic, erosion of the orbital roof	Imaging	Observation	Initial observation: incidental finding, with no associated symptoms
6	M, 8, palpable eyelid mass	1- Dermoid cyst 2- Cavernous venous malformation 3- Rhabdomyosarcoma	1.7x1.9x1.9	Extraconal, superior, medial, anterior, extends into the superior eyelid	Demarcated, homogenous, lobulated, cystic, erosion of the medial orbital wall	Histology	Biopsy and observation for 1.5 years before surgery	1) Initial observation: minimal symptoms, and intricate surgery 2) Subsequent surgery: rapid tumor growth
7	F, 60, pain and discomfort in the orbital region	1- Schwannoma 2- Cavernous venous malformation	2.8x2.0x2.0	Intraconal, posterior, extends to the apex	Demarcated, heterogenous, lobulated, cystic, no erosion	Histology	Biopsy and observation	Minimal associated symptoms, and surgery contraindicated due to proximity to the optic nerve
8	M, 21, palpable eyelid mass	1- Cavernous venous malformation 2- Schwannoma 3- Dermoid cyst	4.0x2.0x1.8	Extraconal, superior, medial, anterior, extends to both; apex posteriorly and superior eyelid anteriorly	Demarcated, homogenous, lobulated, erosion of the roof and lateral wall of the orbit	Histology	Observation for 15 years before surgery	1) Initial observation: refusal of surgery against medical advice 2) Subsequent surgery: extreme tumor growth, pressure on the globe, and severe bony erosion of the roof and lateral wall of the orbit
9	M, 53, hypoglobus	cavernous venous malformation	1.4x1.7x1.6	Extraconal, superior, medial, anterior	Demarcated, heterogenous, cystic, erosion of the frontal bone	Histology	Biopsy and surgery	Displacement of the eye, with significant symptoms
10	F, 85, incidental	1- Lymphoma 2- Cavernous venous malformation	2.0x2.0x2.0	Intraconal, posterior, extends to the apex	Demarcated, heterogenous, cystic, no erosion	Histology	Biopsy and observation	Incidental finding, with no associated symptoms
11	M, 47, reduced visual acuity and proptosis	1- Cavernous venous malformation 2- Schwannoma	4.0x2.0x1.6	Intraconal, posterior, with extension into the cavernous sinus	Demarcated, heterogenous, erosion of the orbital fissure	Histology	Biopsy and observation	Surgery contraindicated due to proximity to the optic nerve, infiltration of cavernous sinus, as well as patient's severe cardiovascular comorbidities
12	F, 74, diplopia and headache	1- Metastasis 2- Malignant peripheral nerve sheath tumor	2.4x1.7x1.8	Intraconal, posterior, with extension into the cavernous sinus	Demarcated, heterogenous, erosion of the orbital fissure	Histology	Biopsy and observation	Minimal associated symptoms, and surgery contraindicated due to proximity to the optic nerve, and extension into cavernous sinus
13	F, 44, incidental	Schwannoma	1.8x1.4x1.1	Extraconal, superior, anterior, extends to the apex	Demarcated, heterogenous, lobulated, cystic, erosion of the orbital roof	Histology	Observation for 1.5 years before surgery	1) Initial observation: Incidental finding, minimal symptoms, and intricate surgery 2) Subsequent surgery: rapid tumor growth, and pressure on the globe
14	M, 84, incidental	1- Schwannoma 2- Cavernous venous malformation	1.3x1.0x1.2	Intraconal, posterior	Demarcated, heterogenous, no erosion	Imaging	Observation	No associated symptoms, as well as advanced patient age
15	F, 62, incidental	Schwannoma	1.3x0.9x0.9	Extraconal, posterior	Demarcated, heterogenous, lobulated, cystic, no erosion	Imaging	Observation	Minimal associated symptoms
16	F, 51, pain and discomfort in the orbital region	Cavernous venous malformation	1.8x1.4x1.8	Intra- & extraconal, posterior	Demarcated, homogenous, no erosion	Histology	Surgery	Pressure on the globe

Initial imaging revealed well-demarcated tumors of varying sizes, often heterogeneous (*n* = 11; 69%) and cystic (*n* = 11; 69%) on MRI. Half of the tumors exhibited lobular growth patterns (*n* = 8). The location of the tumor was extraconal in eight cases (50%), intraconal in six cases (38%), and mixed intra- and extraconal in 2 cases (12%). Extension of the tumor into the skull base was found in five cases (31%), where four passed through the orbital fissure and one through the orbital roof. Thinning of adjacent bone in conjunction with the slow growth of the tumor was seen in 10 patients (63%), involving the superior orbital fissure, the orbital roof, the medial, and lateral walls of the orbit, or the frontal bone (Table 1).

Surgical management was chosen in 4 patients (25%) and conservative management in 12 patients (75%). Among the 12 conservatively managed, later follow-ups resulted in delayed surgery in 3 patients (25%, Table 1).

### Presenting signs and symptoms

Ten patients had symptoms on presentation (63%), the rest of the cases were discovered incidentally. Symptomatic patients reported an average symptom duration of 18 months prior to presentation and diagnosis. Proptosis was the most common finding (*n* = 12; 75%), followed by vertical displacement of the eye globe (*n* = 7; 44%) with all but one exhibiting hypoglobus rather than hyperglobus. Six of the patients (37%) presented with diplopia, five (31%) with pain or discomfort localized to the orbital region, four (25%) with impaired ocular motility and visual impairment, and three (19%) with ptosis. Presenting signs and symptoms were more common among patients who underwent surgery. More than half of the patients (56%) managed conservatively had been incidentally diagnosed with OS, as opposed to only one patient (14%) among those surgically managed (Table 2).

**Table 2:** Presenting signs and symptoms in patients diagnosed with orbital schwannomas

	Whole cohort (*n* = 16)	Conservative (*n* = 9)	Surgery (*n* = 7)*
Signs and symptoms			
Proptosis (%)	12 (75%)	6 (67%)	6 (86%)
Vertical displacement of the globe (%)	7 (44%)	2 (22%)	5 (71%)
Diplopia (%)	6 (37%)	3 (33%)	3 (43%)
Pain or discomfort in the orbital region (%)	5 (31%)	2 (22%)	3 (43%)
Impaired ocular motility (%)	4 (25%)	3 (33%)	1 (14%)
Visual impairment (%)	4 (25%)	2 (22%)	2 (29%)
Ptosis (%)	3 (19%)	1 (11%)	2 (29%)
Incidental finding (%)	6 (37%)	5 (56%)	1 (14%)

^*^Including patients surgically treated after initially being managed conservatively

### Outcomes

**Fig. 1:**
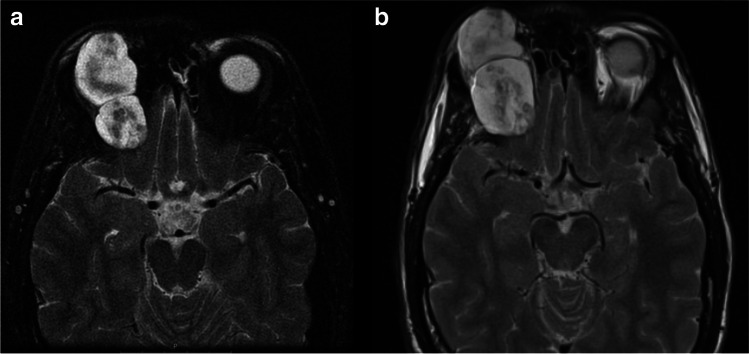
**a**) MRI 5 years after initial diagnosis of a large orbital schwannoma in a patient who had refused surgery (Patient 8), **b**) MRI of the same patient 10 years later showing further growth during the observation period

**Fig. 2:**
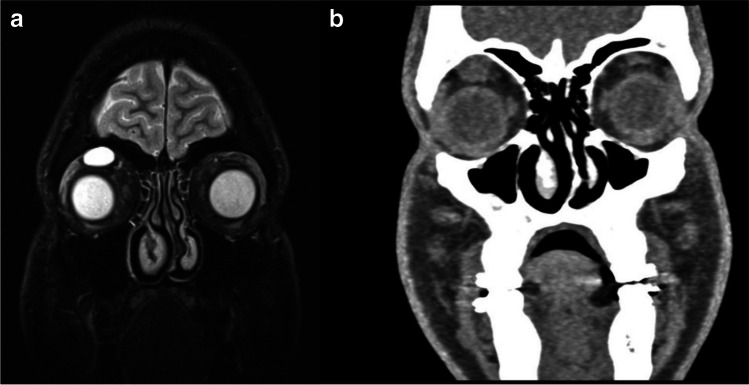
**a**) MRI on presentation supporting the diagnosis of orbital schwannoma (Patient 5), b) CT after 5 years showing no growth of the mass during the observation period

Four patients underwent early surgery (group 1), three with GTR (75%) and one with STR (25%). Three patients, initially treated conservatively, were later operated on with STR, (group 2). One of these three was initially offered surgery due to advanced tumor size and extent of symptoms but had declined. After 15 years of observation, the patient deteriorated due to tumor growth and underwent emergency surgery (Patient 8 in Table 1; and Fig. 1). The two other patients did not experience clinical worsening but were both offered surgery after 1.5 years of observation due to rapid tumor growth, increasing pressure on the eye globe, or thinning of surrounding bony structures (Patients 6 and 13 in Table 2). For groups 1 and 2, the mean postoperative radiological follow-up was 96 and 24 months, while the mean clinical follow-up was 81 and 44 months, respectively. In both groups, no tumor growth or recurrence were detected (two patients lost to radiological follow-up) and all patients had favorable postoperative outcomes characterized by complete symptom resolution (Table 3). One patient operated for an incidentally discovered tumor remained asymptomatic postoperatively. Among the patients conservatively managed (group 3), none experienced any worsening of symptoms at an average of 30 months of clinical follow-up. Moreover, only one of these patients (Patient 12, 11%) experienced tumor growth after 17 months, while the rest had no evidence of further growth over a radiographic follow-up period of 24 months (Table 3, Fig. 2).

**Table 3:** Outcomes in patients with OS depending on the management strategy

	Outcome endpoints	Group 1: Initial surgery (n = 4)	Group 2: Delayed surgery (n = 3)	Group 3: Conservative (n = 9)
Observation period	Radiological outcomes (mean radiographic FU)	n/a	3 (100%) had tumor growth (FU: 67 mos) • Patient 6: 2.2x2.4x2.3cm • Patient 8: 6.3x2.8x1.8cm • Patient 13: 2.0x1.9x1.7cm	8 (89%) had unchanged tumor sizes (FU: 24 mos). 1 (11%) had mild tumor growth (FU: 17 mos) • Patient 12: 2.6x1.6x2.1cm
	Clinical outcomes (mean clinical FU)	n/a	2 (67%) were stable (FU: 20 mos). 1 (33%) experienced worsening (FU: 171 mos): • Patient 8	9 (100%) were stable (FU: 30 mos).
Postoperative period	Extent of resection	3 (75%) GTR and 1 (25%) STR	3 (100%) STR	n/a
	Radiologic outcomes (mean postoperative radiographic FU)	Two patients operated by GTR (67%) had no evidence of recurrence (FU: 135 mos). 1 patient (33%) was lost to FU (Patient 1). 1 patient with STR (100%) had a stable local status (FU: 18 mos).	2 (67%) patients had stable local status (FU: 23 mos). 1 (33%) was lost to FU (Patient 8).	n/a
	Clinical outcomes (mean postoperative clinical FU)	4 (100%) experienced complete resolution of symptoms (median FU: 61 mos)	3 (100%) had favorable outcomes (FU: 44 mos): • 2 experienced complete symptom resolution. • 1 with no initial symptoms (incidental finding) remained stable.	n/a

FU = Follow-up, n/a = Not Applicable

## Discussion

This study reports on a population-based cohort of orbital schwannomas. Of the 16 patients included, four (25%) underwent early surgery at the time of diagnosis, while three were surgically treated later, after initial conservative management (19%), and nine (56%) did not require any surgical intervention during the study period. Although no intraoperative complications occurred in this series, orbital surgery carries the risk of nerve, vessel, and extraocular muscle damage, which may result in both functional and cosmetic sequelae [10, 18]. For patients with no or mild symptoms or who are poor candidates for surgery, the risks of surgical treatment may outweigh the benefits. Conservative management with regular clinical and radiological follow-ups was therefore adopted. Additionally, patients were encouraged to immediately seek care in the advent of any new symptoms or worsening of previous symptoms.

Currently, it is a matter of debate whether GTR should be the gold standard for all patients diagnosed with OS [3]. To date, there are only three cases of conservative management reported in the literature. One case was observed over a 4-year period due to the risk for cosmetic disfigurement in case of surgery. There was no deterioration or increase in tumor size during the observation period [16]. In the second case, surgery was not offered given lack of symptoms and high risk of injury to orbital neurovascular structures. The patient’s neuro-ophthalmic status remained stable at 6 months of follow-up [7]. The third case was an 8-year-old with an orbital schwannoma involving the extraocular muscles. However, outcomes at follow-up were poorly disclosed [4]

Most schwannomas are benign and slow growing WHO grade I tumors. However, intraorbital space is limited and the contained structures are sensitive. To minimize the risk of damage to these structures, surgery is often performed promptly. Surgical tumor removal may come at the cost of injury to the intraorbital structures. Consequently, a conservative management strategy may provide a safer course in selected cases. Of the 12 conservatively managed patients, only three required delayed surgery due to tumor growth (*n* = 3) or worsening of symptoms (*n* = 1). The patient who experienced both tumor growth and worsening of symptoms was initially offered surgery but declined. The rest (*n* = 9) were conservatively managed without requiring further intervention during the study period. Among these patients, only one experienced tumor growth. However, the growth was mild (from 2.4×1.7×1.8 cm to 2.6×1.6×2.1cm) and the patient had no associated symptoms.

Patients presenting with signs of neuro-ophthalmic compromise were offered early surgery. At the study center, STR was often considered when the tumor reached deep into the apex or was adjacent to major neurovascular structures. Of the seven surgically treated patients, four underwent STR (57%) with no reported intraoperative complications, contrary to previous reports [2, 6, 21]. Despite STR, none of these patients experienced tumor growth at 65 months of radiological follow-up. In fact, only two cases of tumor recurrence have been reported in the literature, lending further support to the benign behavior of these tumors.[13] Taken together, this indicates that STR may be sufficient to secure tumor control when GTR may not be safely achievable.

Delaying surgery may arguably do harm through the prolonged compression of intra-orbital structures. However, conservative management allows for surgical intervention when indicated by changes in symptoms or imaging. Nonetheless, this strategy is best suited for tumors that do not compromise the optic nerve or the globe. The group that underwent late surgery had similar outcomes as the early surgical group, demonstrating the success of this strategy.

Radiosurgery, predominantly Gamma Knife surgery (GKS), is increasingly used for benign orbital tumors including schwannomas [20]. However, doses greater than 12 Gy are considered unsafe due to the risk of optic neuropathy and visual acuity impairments are seen with doses ranging from 6 to 16 Gy [14]. Several studies report good tumor control after GKS, however, there are no guidelines for when to pursue GKS over surgical resection [11, 20]. None of the patients in this study were considered for radio surgery, reflecting our own institutional practices. However, radiosurgery may be considered as a viable alternative to surgery or conservative treatment and should incorporated in future treatment guidelines.

**Fig. 3:**
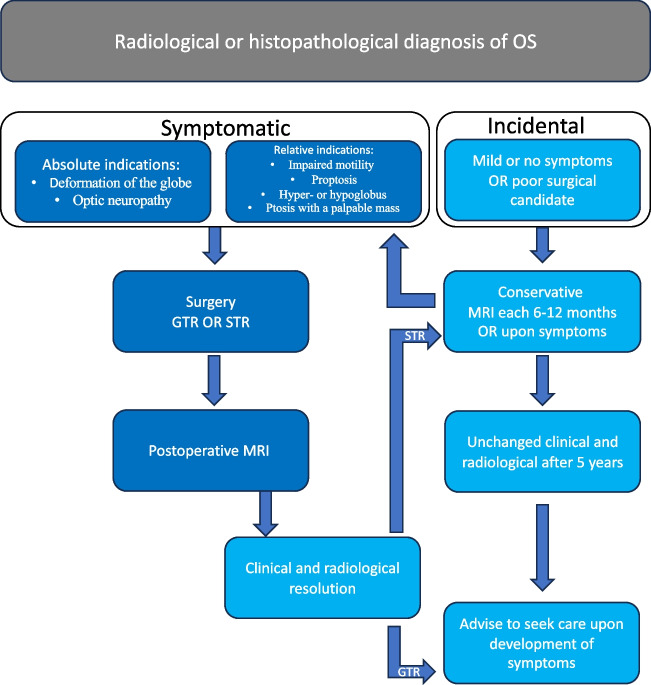
Suggested decision tree for the management of orbital schwannomas. Clinical ophthalmological examination includes visual acuity, pupillary reactions, ocular motility, examination of anterior and posterior segment of the eye, and assessment of globe displacement. For patients with tumor located in the orbital apex or in the vicinity of the optic nerve automated perimetry and OCT is added

In summary, the results of this study suggest that surgery may be avoided or delayed in a selected number of patients presenting with mild or no symptoms. Conservative management, with clinical and radiological follow-ups with gradually increased intervals are therefore advocated in these cases. In cases requiring surgery, STR offers tumor control with less surgical risks compared to GTR. Postoperative yearly radiological controls, for a period of 5 years, are suggested to detect rare cases of tumor growth or recurrence (Fig. 3). Yearly follow-ups are motivated by the rarity of the disease and the risk of permanent visual impairment.

### Strengths and limitations

This population-based study is one of the largest series of reported OS with more than 5 years of follow-up. The retrospective study design and the small sample size, however, hamper the level of evidence. Since patients were referred from different regions, radiological and clinical follow-ups were not always consistent. The diagnosis of schwannoma was not histologically confirmed in four of the patients.

## Conclusion

There were no differences in long term outcome between patients who had been managed with early surgery and those operated later after an initially conservative management. Conservatively treated patients had minimal to no symptoms and remained clinically stable throughout the follow-up period. Based on these findings, conservative management may successfully be adopted in cases with mild symptoms and low risk of neuro-ophthalmic impairment. Conversion to surgical management is indicated upon clinical deterioration or tumor growth.

## Supplementary information

ESM 1 Supplementary file 1: STROBE guidelines checklist. (DOCX 32 kb)
